# The Biological Variation in Serum ACE and CPN/CPB2 Activity in Healthy Individuals as Measured by the Degradation of Dabsylated Bradykinin—Reference Data and the Importance of Pre-Analytical Standardization

**DOI:** 10.3390/proteomes13030040

**Published:** 2025-08-27

**Authors:** Malte Bayer, Michael Snyder, Simone König

**Affiliations:** 1 Core Unit Proteomics, Interdisciplinary Center for Clinical Research, Medical Faculty, University of Münster, Röntgenstr. 21, 48149 Münster, Germany; malte.bayer@ruhr-uni-bochum.de; 2 Knappschaft Clinics University Hospital Bochum, Department of Anesthesiology, Intensive Care Medicine and Pain Therapy, Medical Proteome Center, Medical Faculty, Ruhr University Bochum, Universitätsstr. 150, 44801 Bochum, Germany; 3 Department of Genetics, Stanford University, 450 Jane Stanford Way, Stanford, CA 94305, USA; mpsnyder@stanford.edu

**Keywords:** ACE, serum quality, renin–angiotensin system

## Abstract

Background: Bradykinin (BK) is an inflammatory mediator. The degradation of labeled synthetic BK in biofluids can be used to report on the activity of angiotensin-converting enzyme (ACE) and basic carboxypeptidases N and CBP2, for which the neuropeptide is a substrate. Clinical studies have shown significant changes in the serum activity of these enzymes in patients with inflammatory diseases. Methods: Here, we investigated variation in the cleavage of dabsylated synthetic BK (DBK) in serum and the formation of the major enzymatic fragments using a thin-layer chromatography-based neuropeptide reporter assay (NRA) in a large cohort of healthy volunteers from the international human Personal Omics Profiling consortium based at Stanford University. Results: Four major outcomes were reported. First, a set of NRA reference data for the healthy population was delivered, which is important for future investigations of patient sera. Second, it was shown that the measured serum degradation capacity for DBK was significantly higher in males than in females. There was no significant correlation of the NRA results with ethnicity, body mass index or overnight fasting. Third, a batch effect was noted among sampling sites (HUPO conferences). Thus, we used subcohorts rather than the entire collection for data mining. Fourth, as the low-cost and robust NRA is sensitive to enzyme activity, it provides such a necessary quick test to eliminate degraded and/or otherwise questionable samples. Conclusions: The results reiterate the critical importance of a high level of standardization in pre-analytical sample collection and processing—most notably, sample quality should be evaluated before conducting any large and expensive omics analyses.

## 1. Introduction

### 1.1. Bradykinin

**Figure 1:**
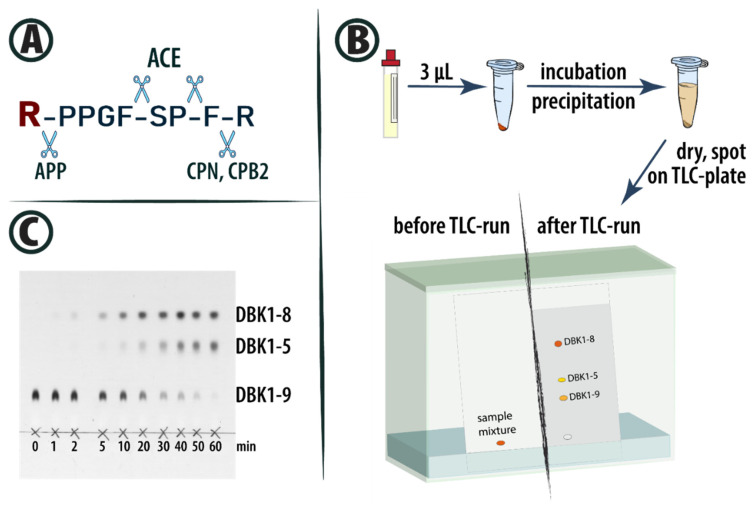
(**A**) Structure of BK and cleavage sites of ACE, basic carboxypeptidases and aminopeptidase P (APP). The dabsylation site at the N-terminus of BK is marked in red. (**B**) NRA workflow using DBK (for more details, see ref. [15]). (**C**) Exemplary TLC plate showing results of incubations of DBK with serum for up to 60 min.

The neuropeptide bradykinin (BK, Figure 1A) is an inflammatory mediator involved in many physiological processes (for BK-mediated signaling networks, see ref. [1]). It is part of the renin–angiotensin system (RAS) and the kininogen–kallikrein system (KKS) and is important in blood pressure homeostasis [2,3,4,5]. The multifunctional KKS forms bioactive kinins and is involved in processes such as blood pressure control, vascular permeability, pain sensation and inflammatory response [6]. The RAS is one of the most potent cardiovascular regulators and is also associated with pro-inflammatory mechanisms and pathological conditions [2,4]. In the recent COVID-19 pandemic, angiotensin-converting enzyme (ACE)-2, which counterbalances the RAS, has drawn much attention as both the receptor for severe acute respiratory syndrome coronavirus 2 (SARS-CoV-2) and a potential therapeutic target [7]. Both RAS and KKS have thus become of increasing interest in the aftermath of the pandemic for their role in the infection process, and it is suggested that they could be predictive for Long COVID [5,8,9]. Even the dysregulation of BK, specifically, has been associated with the pathogenesis of COVID-19 [10]. BK is a substrate of ACE [4,11] and basic carboxypeptidases N (CPN) [12] and B2 (CPB2, also known as thrombin-activatable fibrinolysis inhibitor (TAFI), CPU and pCPB [13]). These enzymes are interconnected in RAS, KKS and other processes such as fibrinolysis (for brief overview, see ref. [14] and citations therein).

### 1.2. Neuropeptide Reporter Assay

Due to its multimodal functionality, the neuropeptide BK is also a target in pain research [16]. For a clinical study with patients suffering from complex regional pain syndrome (CRPS) [17], we developed an assay to monitor the serum enzyme activity using synthetic dabsylated BK (DBK1-9 or DBK for short) [15]. The experiments demonstrated compromised ACE serum activity in this painful inflammatory disease [17,18]. Subsequently, we used the assay in an investigation of sera of COVID-19 patients and noted significantly reduced CPN/CPB2 activity and over-active ACE in severe disease progression [9,19,20]. In recent studies of inflammatory skin diseases, we detected reduced ACE activity in psoriasis (in review).

The workflow of the neuropeptide reporter assay (NRA) is based on thin-layer chromatography (TLC) separation of the enzymatic cleavage products of DBK (Figure 1B) [15]. Three molecules are detected: DBK itself, the cleavage product by ACE (DBK1-5) and the product from basic carboxypeptidase activity (DBK1-8; for exemplary data, see Figure 1C). The assay was initially developed and validated for serum [15,17], but can also be used with capillary blood and dried blood spots, considerably extending its application range [14,21,22]. The NRA can be used to monitor enzyme kinetics, but we were typically more interested in measurements at a given time point.

So far, in our clinical studies, we used comparatively small control groups of either healthy probands and/or patients with diseases of interest. However, NRA results of serum protease activity can potentially have prognostic importance when generated at larger scale. The fact that the assay is low-tech, low-cost and robust bears the promise of a rapid and simple diagnostic test [14]. We were thus interested in data on the natural variability of the NRA results and joined the international consortium “Community-Based Personalized Omics Profiling to Assess the Population’s Omics Variation and Dynamics” (human Personal Omics Profiling, hPOP) based at Stanford University. In this project, biofluids of hundreds of healthy volunteers were studied by other groups using different analytical methods including proteomics and metabolomics [23]. As part of the project team, we measured 340 sera with the NRA. The results demonstrated the robustness and reproducibility of the assay. The data constitute a valuable reference set for the NRA, and support its broader use as a pre-analytical standardization tool.

## 2. Materials and Methods

### 2.1. Sample Cohorts and Permissions

Ethical permission for this study was obtained from both Stanford University (eProtocol #: 34563, IRB 7 registration 5136) and the Ethics Commission of the University of Münster (2020-399-f-S, 20 August 2020). Children younger than 18 years of age, pregnant women and probands suffering from known diseases were excluded. Samples were collected from volunteering scientists attending the HUPO conferences in Boston and Taipei (2016), Dublin (2017) and Orlando (2018) as well as from volunteers recruited in California by the Snyder Lab (2018). Subjects were pseudonymized and, in addition, grouped according to age range by decade (e.g., 20–30 y) and combined ethnicity (e.g., Asian, Caucasian; Table 1, Supplementary Excel Table S1). Body mass index (BMI) and the hours of overnight fasting before sampling were available as physiological parameters [23].

### 2.2. Method

DBK was ordered from Peptide Specialty Laboratories (Heidelberg, Germany); captopril and EDTA were obtained from Sigma-Aldrich (St. Louis, MO, USA). The protease activity of individual samples was visualized by TLC following the incubation of serum with DBK. The assay was performed as described before [15,17] with slight modifications (Figure 1B). Briefly, 500 pmol DBK was incubated with 3 µL serum for 60 min at 37 °C [24]. This time point was chosen for its robustness compared to shorter incubation periods. In healthy subjects, DBK degradation to DBK1-8 and DBK1-5 was nearly complete by then (greater than 90%, see Figure 1C). The reaction was stopped by adding 18 µL ice-cold acetone. Proteins were precipitated overnight at −20 °C. Samples were centrifuged at 20,000× *g* for 1 h and 4 °C. The supernatant was transferred to a new vial and dried under vacuum. The reaction mixture was subsequently resuspended in 1 µL methanol and spotted onto a TLC plate. Vials were washed with 1 µL methanol and this solution was added to the same TLC spot. For inhibition experiments on 38 selected samples from the Taipei group, saturated aqueous EDTA solution (3 µL) or captopril (1 µg) in water were added to the vial containing dry DBK and dried using a speedvac before incubation with serum (for more details on the method, see [18]).

For TLC separation, a mixture of methanol, chloroform, H_2_O and acetic acid (11:4:0.6:0.09, *v*:*v*:*v*:*v*) was used as the mobile phase, and samples were resolved at room temperature. Subsequently, the dry TLC plates were scanned using a conventional flatbed scanner (Epson Expression 1680 Pro, Düsseldorf, Germany). Images were converted to grayscale, and spot intensities were quantified using the software JustTLC v4.03 (Sweday, Sodra Sandby, Sweden). In order to account for any possible losses during sample preparation, as well as plate-to-plate variation, relative values of each spot were calculated by dividing the intensity by the summed intensity of all spots resolved per sample. Each sample was analyzed in triplicate on three different TLC plates to minimize batch effects (three technical replicates). Additionally, on each TLC plate, one sample of NIST reference serum (National Institute of Standards and Technology, Gaithersburg, MD, USA; SRM 909c) previously incubated with DBK was resolved in parallel as a technical control.

IBM SPSS Statistics V. 26 software was used to check for data normality and to carry out *t*-tests. Cohen’s d for effect size was determined by running a *t*-test on the z-scores for the calculation of the mean difference. Further data analyses and visualizations were carried out with R statistical software (v4.3.2).

## 3. Results and Discussion

### 3.1. External Standard

NIST reference serum was incubated with DBK in the same manner as the samples and resolved in parallel with the subject samples. These experiments showed high reproducibility (<3% error for DBK degradation, Supplementary Excel Table S2). The NRA data also indicated that the NIST serum had less than half the activity of serum routinely obtained from healthy persons, likely due to prior treatment and/or the addition of protective constituents by NIST. In fact, this reference material was not prepared nor referenced and advertised for the measurement of enzymatic activity but rather for the analysis of select compounds. Nevertheless, its use with every experiment provided a critical way to assess and confirm assay stability.

### 3.2. Variability in Sample Quality

A major observation in our experiments was a batch effect among collection sites (HUPO conferences). In fact, the results from the first collection differed significantly from those of the other sites and the results for, respectively, two of the other collections (Taipei and Orlando, Dublin and California) were closer together. Sample handling procedures had a high influence on the experimental outcome. Phlebotomy has been recognized as a critical step in clinical research and guidelines were provided by the World Health Organization [25]. Human skill and experience still play a major role in the procedure; red, hemolytic serum can result from drawing blood too fast or from the use of smaller diameters of cannula and needles [26,27]. Differences in training of personnel may thus have played a role, taking into account that sampling has taken place over several years and different persons have been involved.

Hemolysis interferes with many laboratory methods including standard lab tests for lactate dehydrogenase, aspartate aminotransferase and even minerals like potassium [27,28,29,30,31,32,33], because due to erythrocyte destruction, hemolytic samples contain a different molecular composition than regular serum. Color-coded hemolysis charts were published [34,35] to recognize the deep orange to red hemolytic serum samples. In order to avoid the bias of visual inspection [36], spectrophotometry and even deep learning have been proposed to evaluate the degree of hemolysis [28,37,38,39,40,41].

However, using our NRA, we also observed low enzymatic activity for samples, which were not conspicuous based on their color. We had faced this problem before in an unrelated earlier study, where the non-performance of some samples in the NRA originated from the use of different collection tubes by the medical staff [42]. We suggest that the NRA can be a low-cost tool to detect samples of poor quality.

Procedures such as freeze–thaw cycles, exposure to heat and prolonged standing times at room temperature also have a detrimental effect on sample quality due to uncontrolled protein denaturation and/or cleavage [28]. In fact, it was shown that standing times of 2 h at 4 °C already had a significant impact on protein quantities, and the influence on the metabolome was even higher [43].

The present investigation underscores the importance of pre-analytical standardization in order to ensure reproducibility. These issues have been discussed for decades and are still not resolved. However, they are a basic requirement for the widely conducted expensive multi-omics research, which tackles enormous molecular complexity [44,45].

### 3.3. General Observations

**Figure 2:**
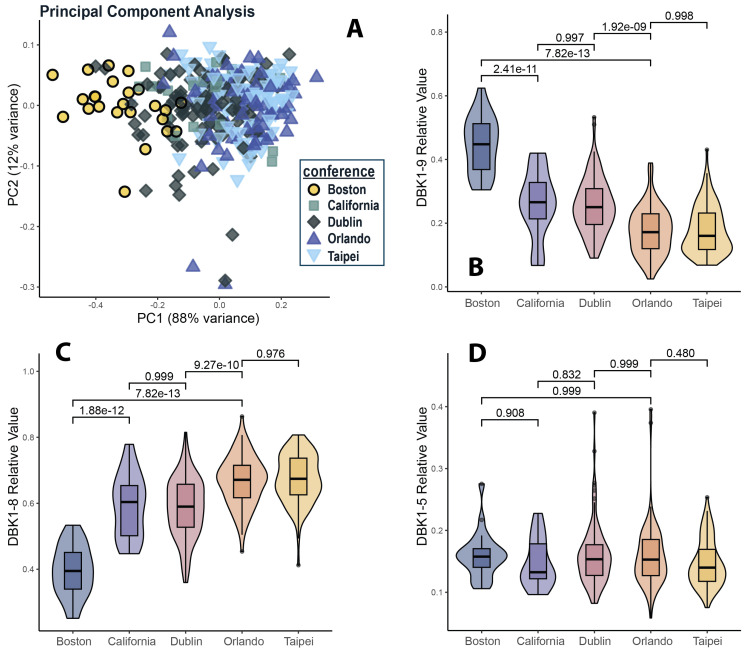
Principal component analysis (PCA) of all non-hemolytic samples, color-coded by sampling location (**A**), and violin boxplots of relative DBK1-9 degradation (**B**) as well as DBK1-8 (**C**) and DBK1-5 (**D**) formation, with corresponding Tukey’s post hoc *p*-values after two-sided ANOVA between the sampling locations.

With the present workflow, DBK was degraded to about one fifth of its original amount. DBK1-8 was the dominant fragment reaching relative intensities of almost 70% while DBK1-5 was formed at only about 15%. The latter fragment was thus, because of the larger error margin, not useful to study small effects in the healthy population, but it was important in the context of pathologies such as CRPS [17] or COVID-19 [9,19,20]. The DBK degradation capacities of all sera are shown in Figure 2 and Table 2 (for details, see Supplementary Figure S1 and Supplementary Excel Table S1). The detected protease activity varied only slightly with sampling event, except for the Boston collection; these samples showed significantly lower neuropeptide processing than those from the other locations (Figure 2). This observation could be explained by the fact that Boston was the pilot stage of the hPOP study and sampling was not fully optimized at this point. These issues were eventually resolved, and the results from the Taipei and Orlando collections were generally consistent with previous assay data [15,17,19]. There was also good overlap with results from the Dublin and California groups, but a higher variance was noted compared to the Taipei and Orlando collections. In particular, for the Dublin collection, the highest share of hemolytic samples was observed. As shown in Supplementary Figure S1, hemolysis negatively affected neuropeptide degradation. In contrast, results for lipidemic samples were not conspicuous with regard to their response in the NRA. After exclusion of those low-quality samples from the analysis, Tukey post hoc *p*-values between the Dublin and Orlando sample sets were highly significant for DBK1-9 and DBK1-8 (1.92 × 10^−9^ and 9.27 × 10^−10^, respectively). For DBK1-5 no batch effect was observed for any of the conference sites, likely due to the fact that it was formed at low quantities (Figure 2).

Taken together, the combined analysis of all samples generated results which were within the range established by previous studies [15,17,19]. However, the inclusion of poor-quality (e.g., hemolytic) samples led to an overall reduced measurement accuracy and increased standard deviation. Moreover, detailed information such as gender-dependency could not be assessed in a statistically significant manner when data from the entire cohort was analyzed, but was feasible for individual collection sites (see Section 3.4, Section 3.5 and Section 3.7). Thus, hemolytic samples were removed from further analyses and subsequent assessments were performed for individual collection events so as to minimize batch effects. A general dependency of protease activity on age was not observed; a slightly reduced activity in females compared to males was, however, noted. The only exception was the California cohort, which was by far the smallest with only 7 female and 11 male subjects. A few extreme values in the data were seen, but reasons for the outliers could neither be elucidated nor validated in the present study. Below, results for the different sampling events are discussed separately.

**Table 3:** Standard deviations and mean average values for sample subsets considering sex. Values useful for future reference are labeled in bold/italic.

	DBK 1-9	Std. Dev DBK1-9	DBK 1-8	Std. Dev DBK1-8	DBK 1-5	Std. Dev DBK1-5
**Male**						
Excluding hemolytic samples	0.193	0.086	0.649	0.089	0.158	0.046
***Taipei 2016***	***0.159***	***0.062***	***0.688***	***0.068***	***0.153***	***0.037***
Orlando 2018	0.161	0.072	0.679	0.073	0.160	0.047
**Female**						
Excluding hemolytic samples	0.234	0.089	0.618	0.092	0.148	0.045
***Taipei 2016***	***0.209***	***0.082***	***0.653***	***0.077***	***0.138***	***0.038***
Orlando 2018	0.215	0.067	0.633	0.016	0.153	0.061

### 3.4. Subset Analysis—Taipei

**Figure 3:**
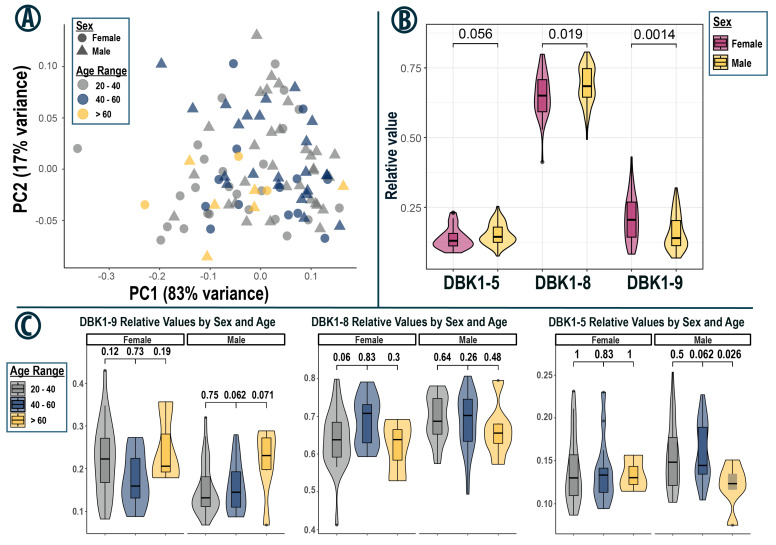
(**A**) PCA of all non-hemolytic samples from the Taipei cohort. The corresponding age ranges are color-coded, with samples from female participants represented by triangles and from males by circles. (**B**) Violin boxplots of the resulting relative DBK1-5 and DBK1-8 formation and DBK1-9 degradation, separated by sex. Welch’s *t*-test *p*-values between males and females are shown above each comparison. (**C**) Violin boxplots of relative DBK1-5 and DBK1-8 formation as well as DBK1-9 degradation for sex and age range. Tukey’s HSD *p*-values after two-sided ANOVA are displayed above each comparison.

The Taipei sample set had the highest number of participants and the least variation. It, therefore, served as an excellent reference set, enabling the analysis of subgroups of test subjects (Figure 3). DBK1-9 degradation and DBK1-8 formation were statistically significantly different between females and males (DBK1-5 formation: Welch’s *t*-test *p*-value = 0.056). There were indications of age dependency; however, only the DBK1-5 formation in males aged 40–60 compared to those over 60 showed a significant difference with a *p*-value of 0.026 (23 vs. 6 participants). Test results were not significantly different with regard to ethnicity, BMI or fasting time prior to sample collection.

### 3.5. Subset Analysis—Orlando

**Figure 4:**
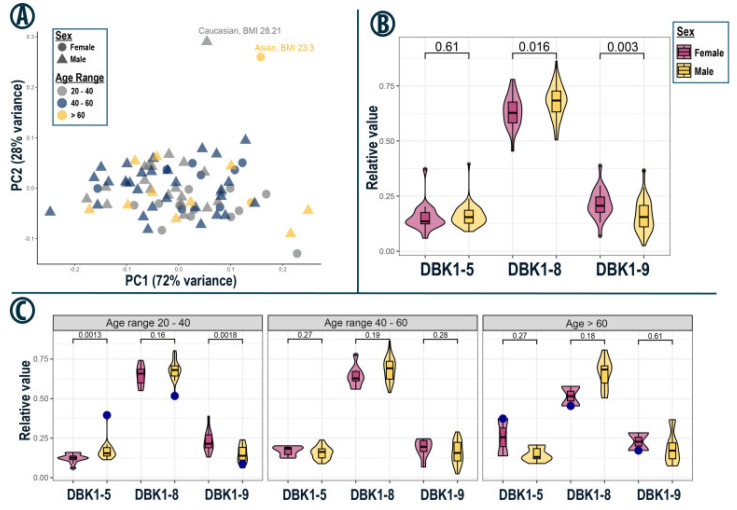
(**A**) PCA for the Orlando set. Females are represented by circles, and males by triangles. Age ranges are color-coded. Two outliers with lower DBK1-8 and higher DBK1-5 formation are labeled with ethnicity and BMI. (**B**) Violin plots of gender-specific DBK1-5 and DBK1-8 formation as well as DBK1-9 degradation. Welch’s *t*-test *p*-values are indicated above each comparison. (**C**) Same as (**B**) for age-dependent subgroups. Outliers from A are marked with blue dots.

The data from the Orlando set are visualized in Figure 4. The formation of DBK1-8 and DBK1-5 increased with high DBK degradation (i.e., low detected values for residual DBK); the formation of the shorter DBK1-5 fragment was generally lower than that of DBK1-8 [17,19]. Two outliers were noted, which were characterized by normal DBK degradation, but low formation of DBK1-8 and unusually high formation of DBK1-5. These values indicated enzymatic dysregulation. One sample came from a middle-aged (age range 30–40 y) Caucasian male who had a stomach virus with fever three weeks before sampling. The other sample originated from an older Asian female (60–70 y) possibly suffering from age-related diseases. Sex-related, statistically significantly reduced DBK1-8 formation and DBK1-9 degradation were confirmed in this data-set as well. DBK1-5 formation, however, was not significantly different between females and males, consistent with the findings in the Taipei subgroup.

The same trend was observed in age-dependent subgroups (Figure 4C), although there, a statistically significant difference in DBK1-5 formation between females and males aged 20–40 could be identified (11 female vs. 20 male subjects). Interestingly, even exclusion of the outlier still yielded a *p*-value of 0.002, indicating that there may have been a sex-specific difference in DBK1-5 formation, although not as distinct as for the other two analytes. This observation is also corroborated by the fact that, for the Taipei cohort, the *p*-value of the DBK1-5 comparison was close to the chosen statistical cutoff of 0.05 (*p* = 0.056, Figure 3B). The Orlando cohort was the collection with the biggest difference in the ratio of males and females (26.5% females); thus testing power was reduced. This was notably the case for subjects over 60 years of age (2 vs. 10). As the general data spread was similar to that of the Taipei set, results from both collections were combined to test for ethnicity-based differences in protease activity, but none were identified (Supplementary Figure S2). Of note, the combination of both cohorts yielded a significant age-related increase in variance in DBK1-9 degradation capacity for male subjects with a Levene test *p*-value of 0.042 (Supplementary Figure S3).

### 3.6. Subset Analysis—Dublin/California

In contrast to the Taipei and Orlando subsets, data variance was higher in the Dublin and California subgroups (Figure 2, Supplementary Figure S4). Due to their similar data spread (Supplementary Figure S5) and the small sample size in the latter, they were combined for statistical analysis. In the Dublin group, three outliers were identified, which were not as distinctive as those in the Orlando set, but still fell outside the bulk data. A Caucasian male (age range 50–60 years) was slightly overweight, but this did not explain the lower DBK1-8 and higher DBK1-5 values measured in his serum. Another outlier originated from an overweight Caucasian male (60–70 years), who was diagnosed with diabetes after the Dublin conference and who took blood-pressure-regulating medication and statins against high cholesterol. This proband attended the Taipei conference the year before and the Orlando conference the year after and there, the measurements were not conspicuous. It can be speculated that his diabetes was just developing in 2016 and was treated in 2018, but more data would be needed to confirm the hypothesis of ACE dysregulation in untreated diabetes. Still, protective effects of ACE inhibitors in diabetes for heart and kidney have been reported [46]. A sample from a fit female (30–40 years), who ran 20 miles per week, regularly participated in marathons and identified as having a healthy diet, also showed deviating NRA results. It is known that strenuous exercise such as long-distance running causes metabolic adaptation [47] and that excessive training (e.g., for a multi-stage ultramarathon of 4500 km [48]) can substantially increase energy metabolism. This outlier thus also deserves further investigation.

### 3.7. Statistical Considerations

In the Orlando, Taipei and the Dublin/California cohorts, data for DBK1-9 and DBK1-8 separated by sex were normally distributed according to both the Kolmogorov–Smirnov and the Shapiro–Wilk test (significance value < 0.05; exceptions of DBK1-9, males, Taipei). For Orlando and Taipei, but not for Dublin/California, the differences for DBK1-9 and DBK1-8 data between males and females were statistically significant (Figure 2 and Figure 3; Table 3). The combined statistical analysis of the samples of all collection events except Boston and the hemolytic samples was not advisable as the data were not normally distributed. The *t*-test nonetheless identified statistically significant differences between males and females. No statistically significant relationships were detected with respect to age, ethnicity, BMI or overnight fasting period.

The cross-tables shown in Supplementary Figure S6 for the Taipei subset indicate a higher DBK degradation capacity at a younger age (<50 y), especially in males, that peaks in the 30–40 y age range. In addition, slightly less protease activity was suggested in sera of Asian compared to Caucasian females at this sampling event (Supplementary Figure S7). The gender-separated cross-tables for age and ethnicity and the sample numbers regarding ethnicity, age and BMI are given in the Supplement (Figures S8–S11). More Asian than Caucasian (58% vs. 37%) women were present in Taipei and they were mainly younger (21 Asian vs. 15 Caucasian females, 20–50 y). There was also a trend among females for higher serum protease activity with lower BMI.

The same questions were asked for the Orlando data (Supplementary Figures S12–S17) and trends partially went in the same direction, but more Caucasians than Asians (64% vs. 27% females) attended this conference so there was limited comparability. There was also no significant bivariate correlation for the combined samples for females of both events with respect to ethnicity or age.

### 3.8. ACE-Specific Inhibition

We performed experiments adding the ACE-specific inhibitor captopril or the metal-chelating agent EDTA to the incubation mix (Supplementary Excel File, Table S3). The latter almost completely abolished all measured enzyme activity while captopril selectively eliminated DBK1-5 formation. Interestingly, there appeared to be a drop in the formation of DBK1-8 for females older than 40 years of age (*p* = 0.001, Supplementary Figure S18). For persons 50 years and older, there also were significant differences between males and females for DBK1-9 and DBK1-8 (*p* = 0.045 and 0.040). It was reported that ACE levels increased with age in females, because estrogen downregulates ACE; after menopause, women have comparable ACE levels to age-matched men [49]. In post-pubescent children and young adults, ACE is lower in females than in males. We do not have an explanation for how ACE-specific inhibition exactly affected the activity of basic carboxypeptidases; further studies with specific inhibitors are necessary to shed more light on this issue.

### 3.9. Limitations

As a result of the batch effect discussed above and smaller sample sizes in separate cohorts, the power of the sub-analyses was reduced. For the NRA, the Taipei collection still represents a sufficiently large sample set for reference purposes. Importantly, this study underlines the critical need for pre-analytical standardization, which is especially true for large scale, multi-center omics projects.

This study is also limited by the available medical information. The hPOP initiative targeted the healthy population and aimed at the analysis of omics data. With the present work, we have taken advantage of this sample collection, but it is not originally designed for use with the NRA. It is thus not known if ACE inhibitors were taken by some probands. Such medication would affect ACE activity and, normally, these samples would be excluded from the study.

## 4. Conclusions

This study is the first investigation of a large cohort of apparently healthy volunteers to assess the degradation of the labeled synthetic neuropeptide BK by serum enzymes. It provides hundreds of experimental measures for both the cleavage of the peptide and the formation of the two major enzymatic fragments; the data thus highlight the natural variation in these parameters in the general population. Comparative experiments in clinical research typically only use a limited number of controls so this larger data-set is an important reference.

Mining of this data-set showed that the detected serum protease activity was statistically significantly higher in males than in females. This was not surprising, because the female hormonal cycle and endocrinological status do affect several enzymatic pathways [49,50,51]. Higher protease activity was also indicated for probands younger than 50 y, especially in males, consistent with the knowledge that aging affects the RAS [52]. Furthermore, the slightly reduced DBK degradation observed in Asian compared to Caucasian females at the Taipei conference may suggest a correlation with nutrition (e.g., phyto-estrogen-containing soy-based vs. meat-based diets [53]) worthy of follow-up.

At the technical end, unexpectedly, we noted inconsistent results among sampling sites largely resulting from pre-analytical factors. Sera from the first sampling event at the Boston HUPO conference differed markedly from the rest and were ultimately excluded from the study. In these early days of hPOP, sampling was not fully standardized and this was improved for the later collections. Thus, we used subcohorts rather than the entire collection for data mining. We did not attempt data normalization to improve matching, because the variation was clearly not due to systematic errors, which could be factored in, but rather to human performance even when following standard sampling protocols. Our results highlight and reiterate the importance of strong and consistent standardization in pre-analytical sample collection and processing. Clearly, before carrying out any large and expensive omics experiments, sample quality should be critically evaluated. Not only are such larger and detailed analyses quite expensive, but they assess enormously complex sets of molecules (e.g., proteoforms [44]), which is a major challenge in itself. Best practices for the contributing laboratories in a multi-center study involve the following: (1) agreement on a standardized workflow from sample collection all the way through to bioinformatic data mining (including choice of manufacturers of chemicals/substances, instrumentation, software), (2) comprehensive training of all personnel (study nurses, students) ensuring sufficient skill, (3) fast communication channels to find joint solutions for observations and arising problems and (4) appropriate data analysis observing limitations and avoiding overinterpretation.

We found that our low-cost and robust NRA is useful as a quick test to weed out degraded samples. It delivers comparatively simple, yet sensitive, information about serum quality. While it can only sensibly be used for the samples of healthy controls (values for patients may vary considerably), problems with them may indicate difficulties in the entire cohort. Thus, the NRA can serve as a critical screening tool before expensive high-tech, big-data, omic analyses are carried out, better ensuring consistent and representative data. Considerable resources could be saved in this way, while protecting data integrity.

In summary, four major outcomes are reported. First, a set of NRA reference data for the healthy population is delivered. Second, it is shown that the measured serum degradation capacity for DBK is significantly higher in males than in females. Third, a batch effect is noted among sampling sites stressing again the need for pre-analytical standardization. Fourth, the NRA provides a quick test to eliminate degraded and/or otherwise poorly handled samples.

## Figures and Tables

**Table 1:** Overview of serum samples of hPOP probands recruited at HUPO conferences and locally in California. Ethnicity was separated into three groups: Caucasian, Asian (including South-Asian Indians) and BL/HO (including Black, Latino/Hispanic and Other). Meta-information was not shared by all subjects. Visual evaluation detected hemolytic (reddish) and lipemic (turbid) samples [23].

		Boston 2016	Taipei 2016	Dublin 2017	Orlando 2018	California 2018
Code #		1	2	3	4	5
**Subjects**		24	105	110	83	18
**Gender**	**Female**	10	41	44	22	7
	**Male**	14	62	66	61	11
**Ethnicity**	**Asian**	6	50	17	22	1
	**Caucasian**	18	51	84	56	17
	**BL/HO**	-	4	9	5	
**Age range**	**20–40**	12	59	57	34	1
	**40–60**	8	37	42	37	14
	**>60**	4	9	11	12	3
**BMI**	**<25**	15	34	62	37	12
	**25–30**	8	15	31	35	5
	**30+**	1	5	17	10	1
**Samples**	**Hemolytic**	-	1	7	1	-
	**Lipemic**	1	-	3	3	-

**Table 2:** Standard deviations and mean average values for NRA results in sample subsets. Outliers (hemolytic samples and Boston cohort) are marked in dark gray and values corresponding to subsets with best results and low standard deviations (Taipei, Orlando) in light gray. Reference values for future studies are labeled in bold/italic (sex not considered here; see Table 3 for that).

	DBK 1-9	Std. Dev DBK1-9	DBK 1-8	Std. Dev DBK1-8	DBK 1-5	Std. Dev DBK1-5
***All samples***	***0.231***	***0.112***	***0.613***	***0.117***	***0.156***	***0.047***
***Excluding hemolytic samples***	***0.227***	***0.109***	***0.619***	***0.110***	***0.154***	***0.045***
Hemolytic samples	0.387	0.120	0.388	0.145	0.225	0.063
Boston 2016	0.445	0.088	0.395	0.077	0.160	0.036
Dublin 2017	0.254	0.086	0.587	0.090	0.159	0.049
California 2018	0.261	0.106	0.592	0.095	0.147	0.039
***Taipei 2016***	***0.180***	***0.074***	***0.674***	***0.073***	***0.146***	***0.038***
Orlando 2018	0.175	0.074	0.667	0.076	0.158	0.050

## Data Availability

Data are available in the Supplementary Materials and at https://doi.org/10.17879/54918735043 and https://doi.org/10.17879/13988723759.

## Supplementary Materials

The following supporting information can be downloaded at: https://www.mdpi.com/article/10.3390/proteomes13030040/s1, Data are publicly available at https://doi.org/10.17879/54918735043 and https://doi.org/10.17879/13988723759.

## Abbreviations

The following abbreviations are used in this manuscript:

ACE	Angiotensin-converting enzyme
ACE2	Angiotensin-converting enzyme 2
AD	Atopic dermatitis
Ang II	Angiotensin II
ANOVA	Analysis of variance
APP	Aminopeptidase P
AT1R, AT2R	Angiotensin receptor 1 and 2
BK	Bradykinin
BMI	Body mass index
COVID-19	Coronavirus disease 2019
CPN, CPB2	Carboxypeptidases N and B2
CRPS	Complex Regional Pain Syndrome
DBK or DBK1-9	Dabsylated bradykinin
DBK1-5, DBK1-8	Enzymatic fragments of DBK
HEPES	2-(4-(2-Hydroxyethyl)-1-piperazinyl)-ethansulfonic acid
hPOP	human Personal Omics Profiling, international consortium “Community-Based Personalized Omics Profiling to Assess the Population’s Omics Variation and Dynamics”
HUPO	Human Proteome Organisation
KKS	Kinin–kallikrein system
NIST	National Institute for Standards and Technology
NRA	Neuropeptide reporter assay
PCA	Principle components analysis
RAS	Renin–angiotensin system
SARS-CoV-2	Severe acute respiratory syndrome coronavirus 2
Std. Dev.	Standard deviation
TLC	Thin-layer chromatography
