# Supplementary material for: The Biological Variation in Serum ACE and CPN/CPB2 Activity in Healthy Individuals as Measured by the Degradation of Dabsylated Bradykinin—Reference Data and the Importance of Pre-Analytical Standardization
Source: Proteomes. 2025 Aug 27;13(3):40. doi: 10.3390/proteomes13030040 (PMC12452422; doi:10.3390/proteomes13030040)
Supplement: Supplementary file 1 [file proteomes-13-00040-s001.zip › Supplement.pdf]

The biological variation in serum ACE and CPN/CPB2 activity in healthy individuals as measured by the degradation of dabsylated bradykinin – Reference data and the importance of pre-analytical standardization

Malte Bayer[1,2], Michael Snyder[3], Simone König[1]*

[1] Core Unit Proteomics, Interdisciplinary Center for Clinical Research, Medical Faculty, University of Münster, Germany
[2] Department of Anesthesiology, Intensive Care Medicine and Pain Therapy, University Hospital Knappschaftskrankenhaus Bochum, Germany & Medical Proteome Center, Medical Faculty, Ruhr University Bochum, Germany
[3] Department of Genetics, Stanford University, CA, USA

*Corresponding author: koenigs@uni-muenster.de

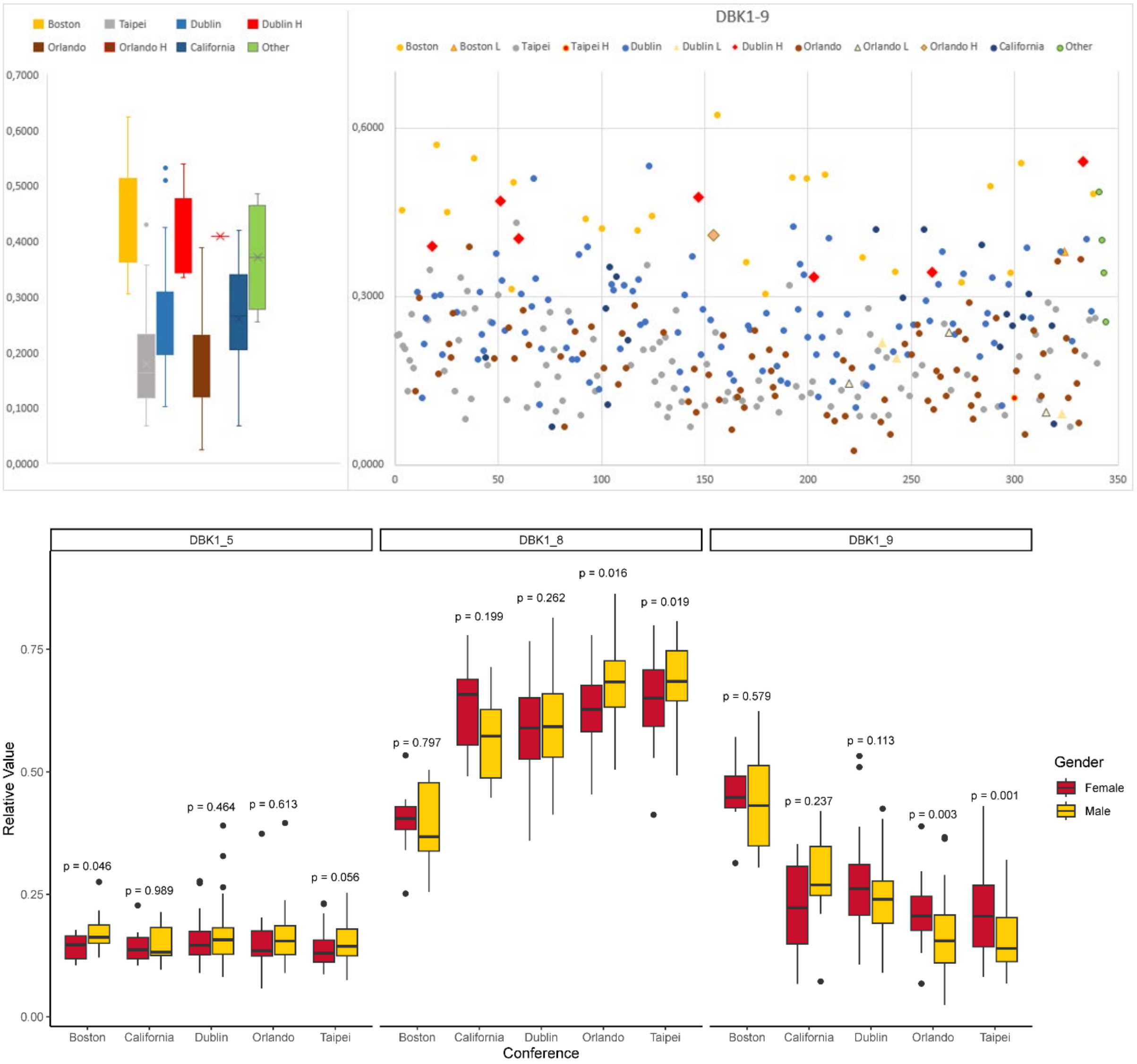

Figure S1: DBK1-9 degradation (relative mean value and corresponding box plot) in 1 h by serum of all healthy volunteers labelled according to sampling event (top). Haemolytic (H) and lipaemiac (L) samples were marked. Visualisation of relative values by conference, gender and peptide without hemolytic samples (bottom). T-test p-values between the gender comparisons are denoted above the boxplots.

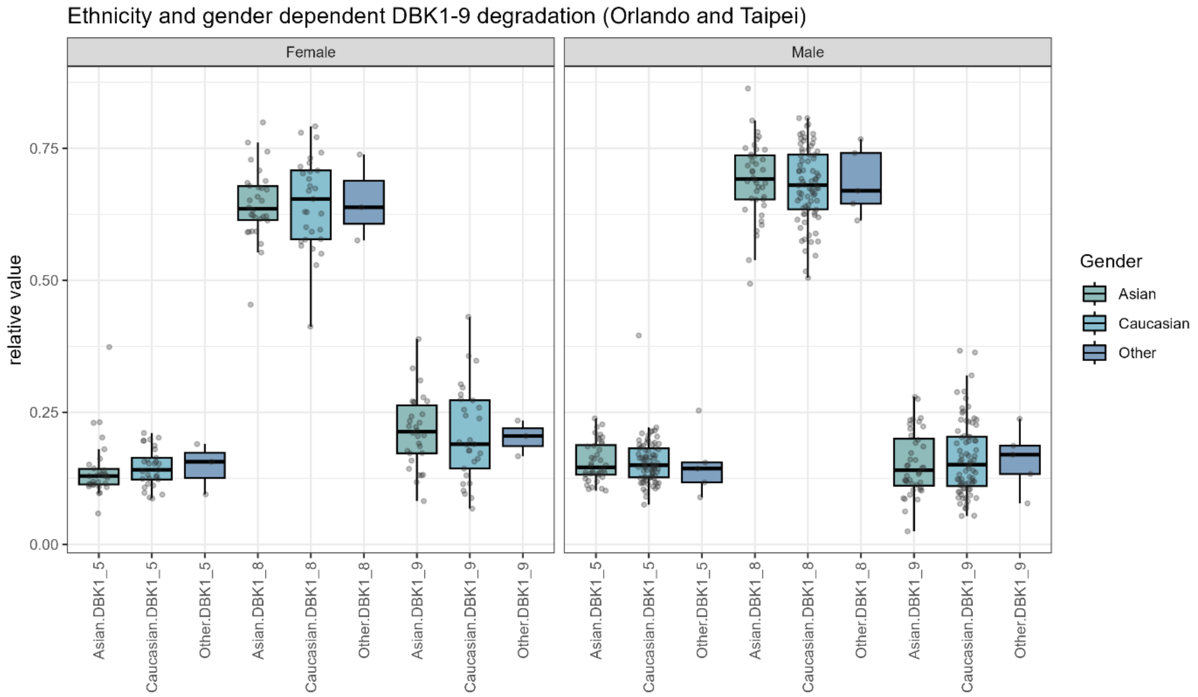

Figure S2: Gender- and ethnicity-separated boxplots of DBK1-9 degradation as well as DBK1-5 and DBK1-8 formation for the combined Orlando and Taipei subsets. Each dot corresponds to the respective value of a subject.

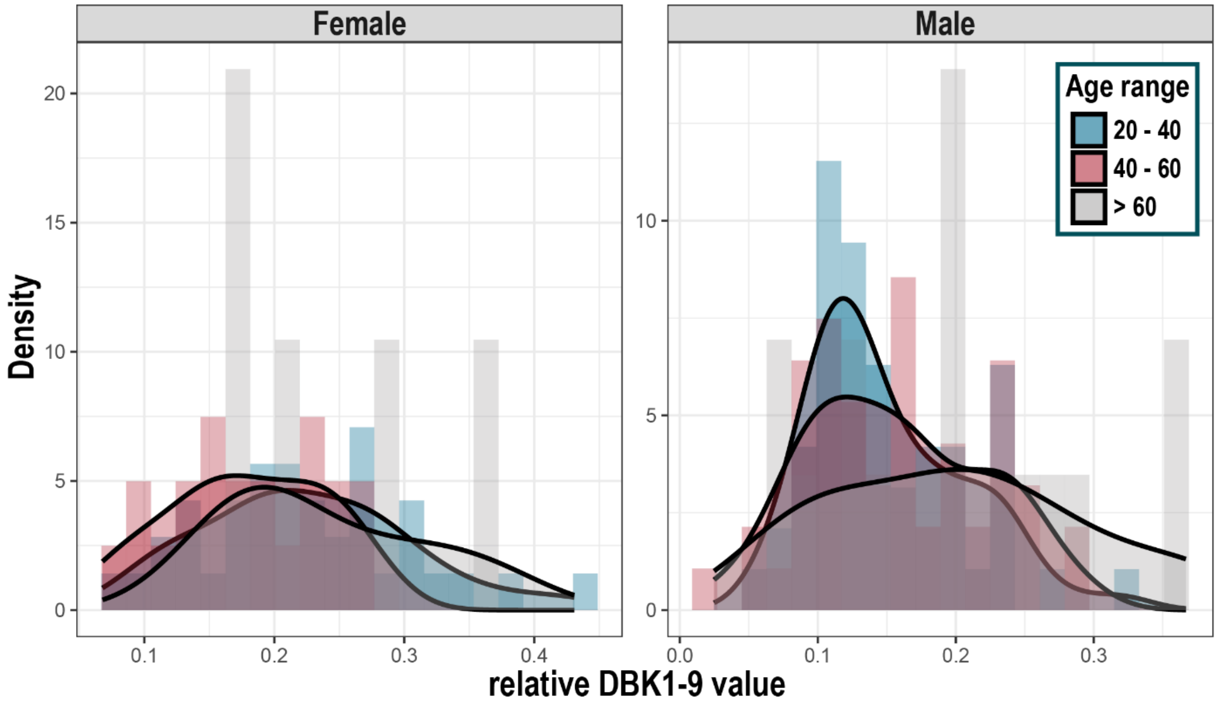

Figure S3: Histogram and density plot of the relative DBK1-9 values of the combined Orlando and Taipei cohorts. Levene test for homogeneity of variances yielded a p-value of $p = 0.573$ for females and $p = 0.04174$ for males.

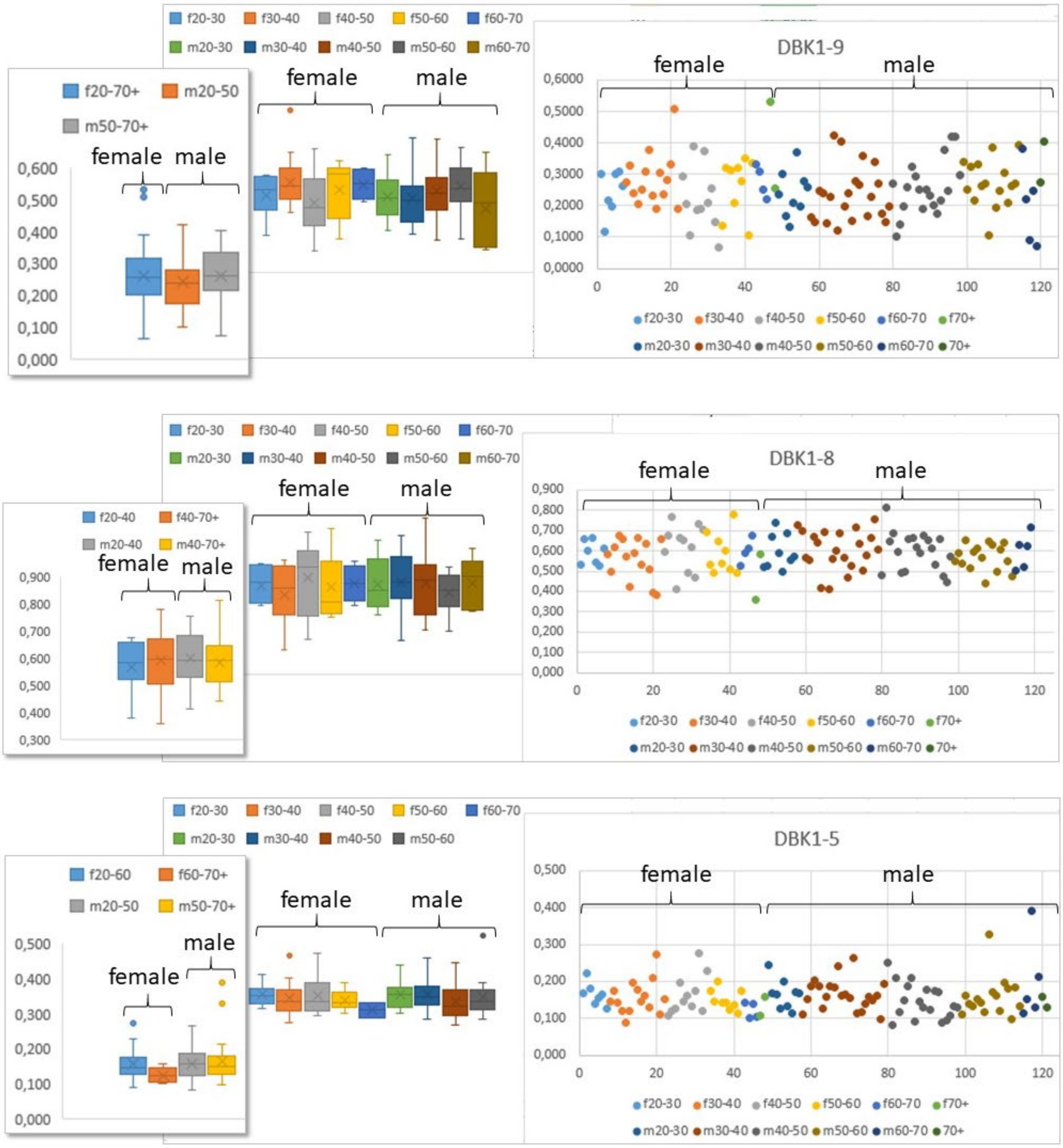

Figure S4: DBK1-9 degradation and DBK1-8 and 1-5 formation results (relative mean intensity, haemolytic samples excluded) for the Dublin / California subset visualized with respect to gender and different age ranges.

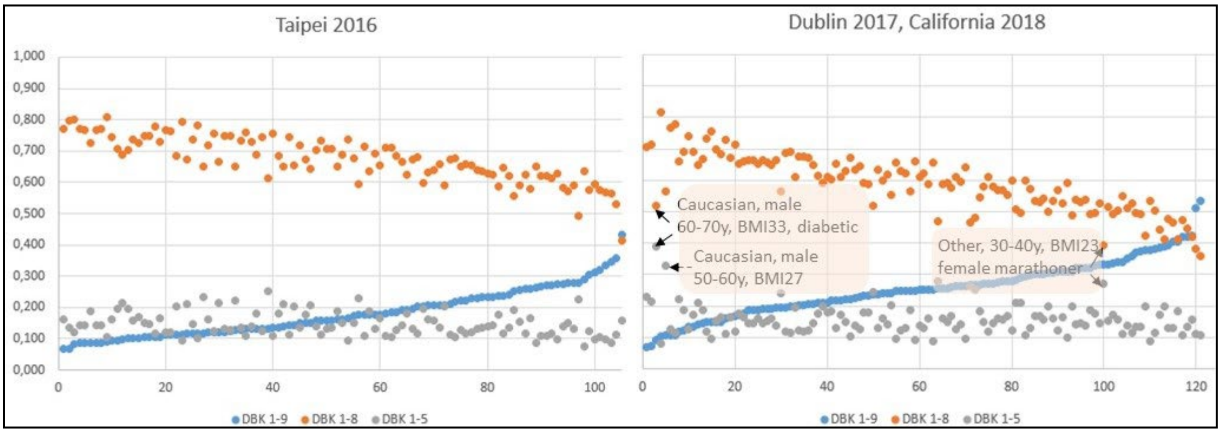

Figure S5: DBK degradation and DBK1-8 and 1-5 formation results (relative mean intensity, haemolytic samples excluded) for the Taipei and combined Dublin / California subsets sorted according to DBK1-9. Three outliers with lower DBK1-8 and higher DBK1-5 production were detected and labelled (transparent boxes).

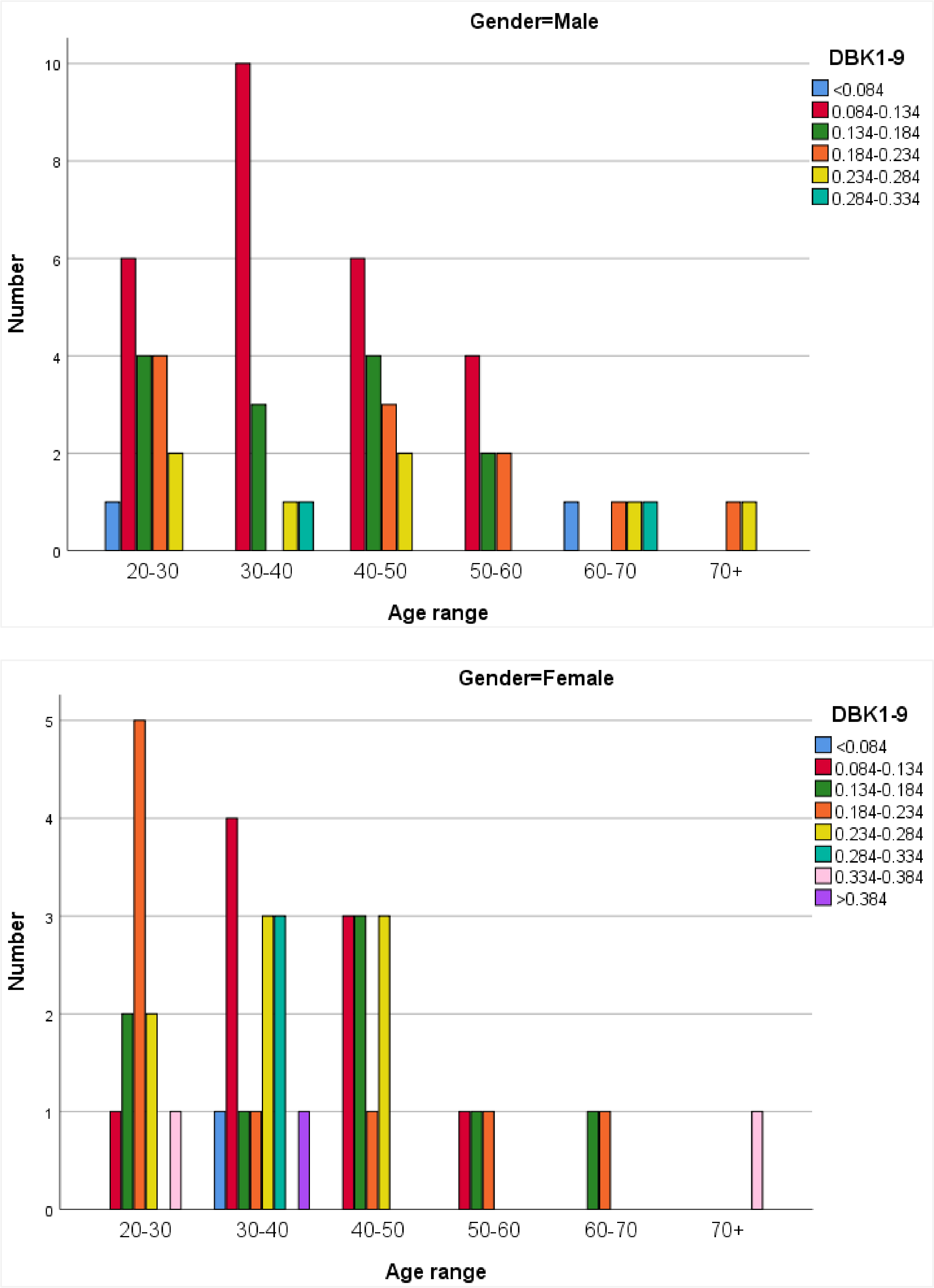

Figure S6: Gender-separated cross-tables of age and DBK1-9 ranges for the Taipei subset.

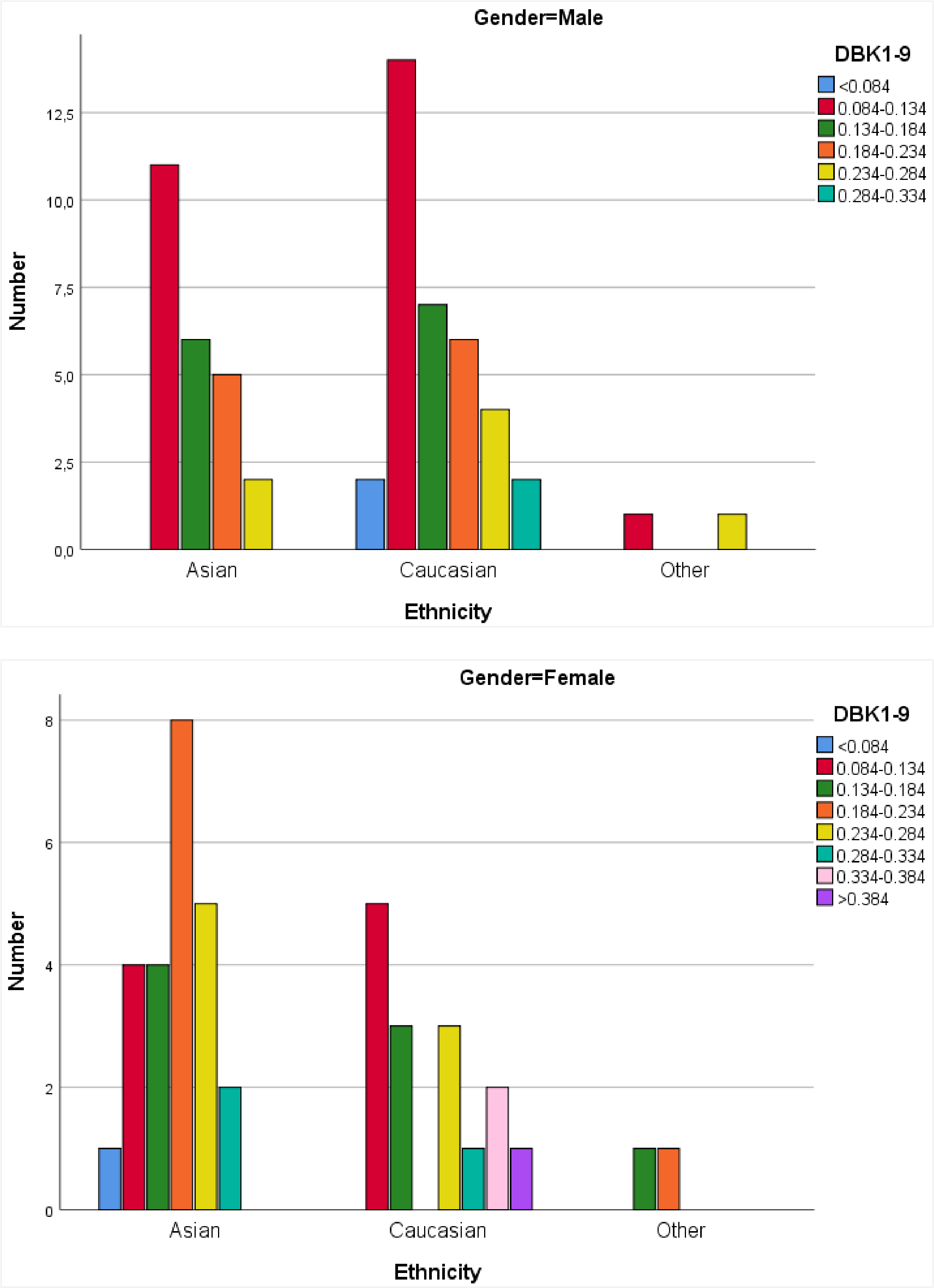

Figure S7: Gender-separated cross-tables of ethnicity and DBK1-9 ranges for the Taipei subset.

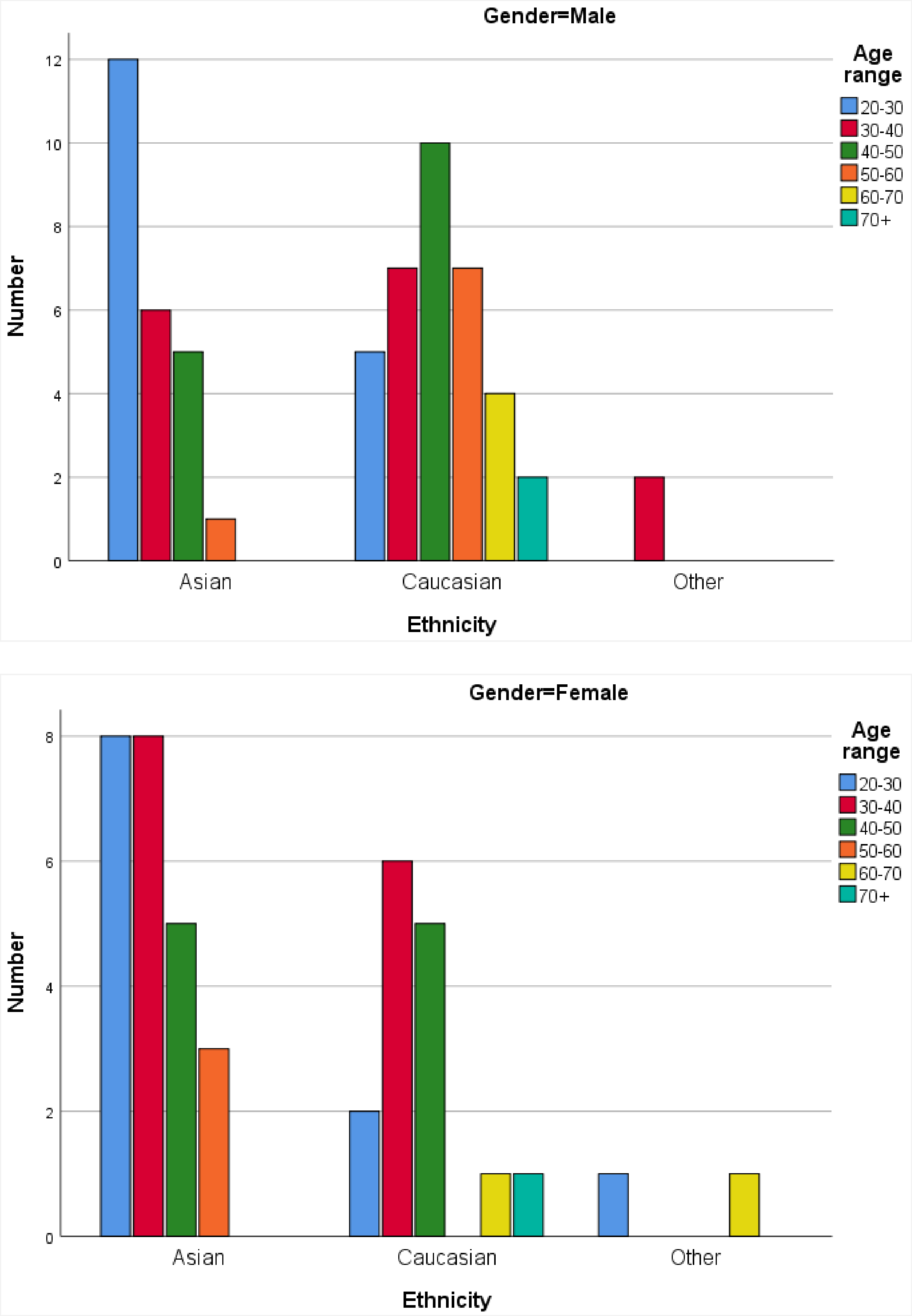

Figure S8: Gender-separated cross-tables of age and ethnicity for the Taipei subset.

Ethnicity

Gender			Frequency	Percentage	Valid percentages	Cumulative percentages
.	Valid	Asian	2	66,7	100,0	100,0
	Missing	System	1	33,3		
	Total		3	100,0		
Male	Valid	Asian	24	39,3	39,3	39,3
		Caucasian	35	57,4	57,4	96,7
		Other	2	3,3	3,3	100,0
		Total	61	100,0	100,0	
Female	Valid	Asian	24	58,5	58,5	58,5
		Caucasian	15	36,6	36,6	95,1
		Other	2	4,9	4,9	100,0
		Total	41	100,0	100,0	

Figure S9: Gender-separated sample numbers regarding ethnicity for the Taipei subset.

Age range

Gender			Frequency	Percentage	Valid percentages	Cumulative percentages
.	Valid	20-30	1	33,3	50,0	50,0
		30-40	1	33,3	50,0	100,0
		Gesamt	2	66,7	100,0	
	Missing	System	1	33,3		
	Total		3	100,0		
Male	Valid	20-30	17	27,9	27,9	27,9
		30-40	15	24,6	24,6	52,5
		40-50	15	24,6	24,6	77,0
		50-60	8	13,1	13,1	90,2
		60-70	4	6,6	6,6	96,7
		70+	2	3,3	3,3	100,0
		Total	61	100,0	100,0	
Female	Valid	20-30	11	26,8	26,8	26,8
		30-40	14	34,1	34,1	61,0
		40-50	10	24,4	24,4	85,4
		50-60	3	7,3	7,3	92,7
		60-70	2	4,9	4,9	97,6
		70+	1	2,4	2,4	100,0
		Total	41	100,0	100,0	

Figure S10: Gender-separated sample numbers regarding age ranges for the Taipei subset.

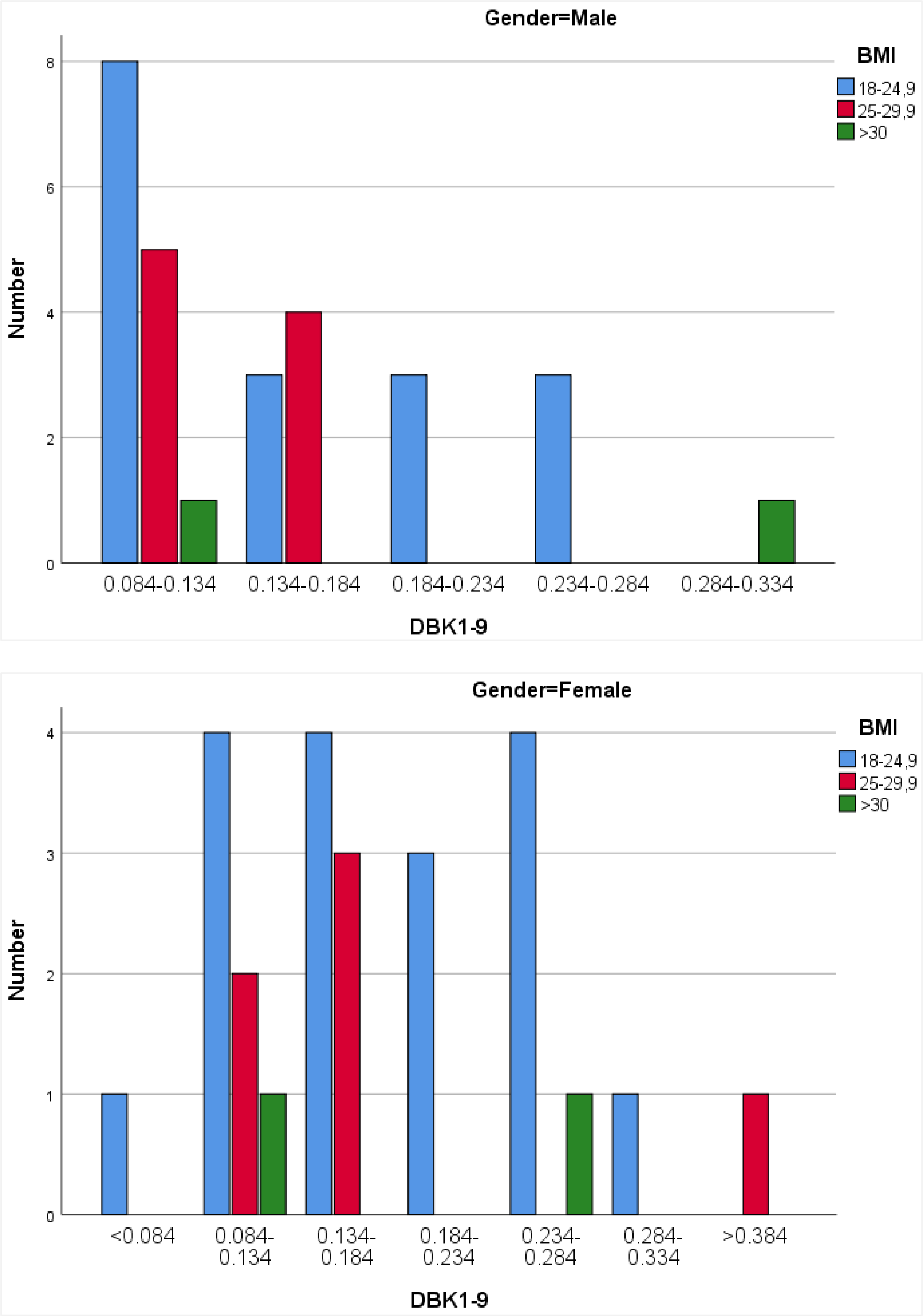

Figure S11: Gender-separated cross-tables of DKB1-9 and BMI for the Taipei subset.

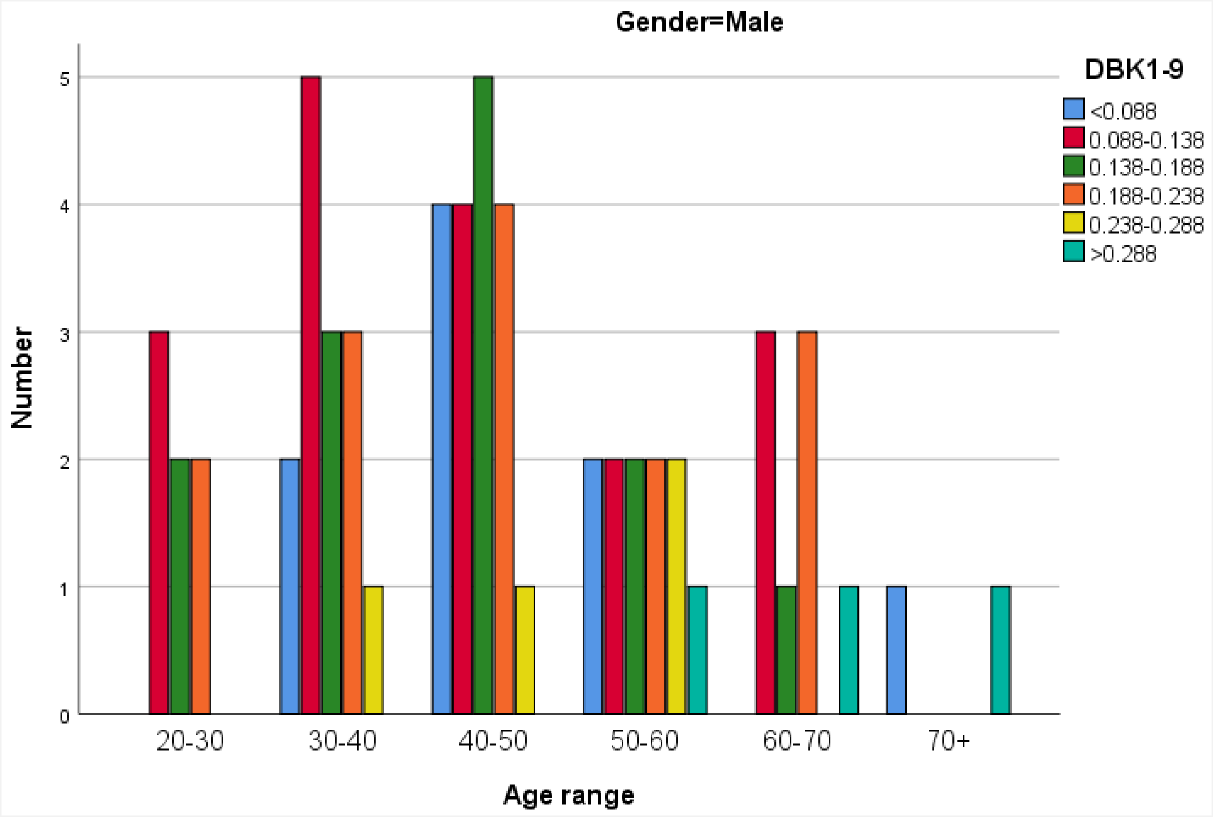

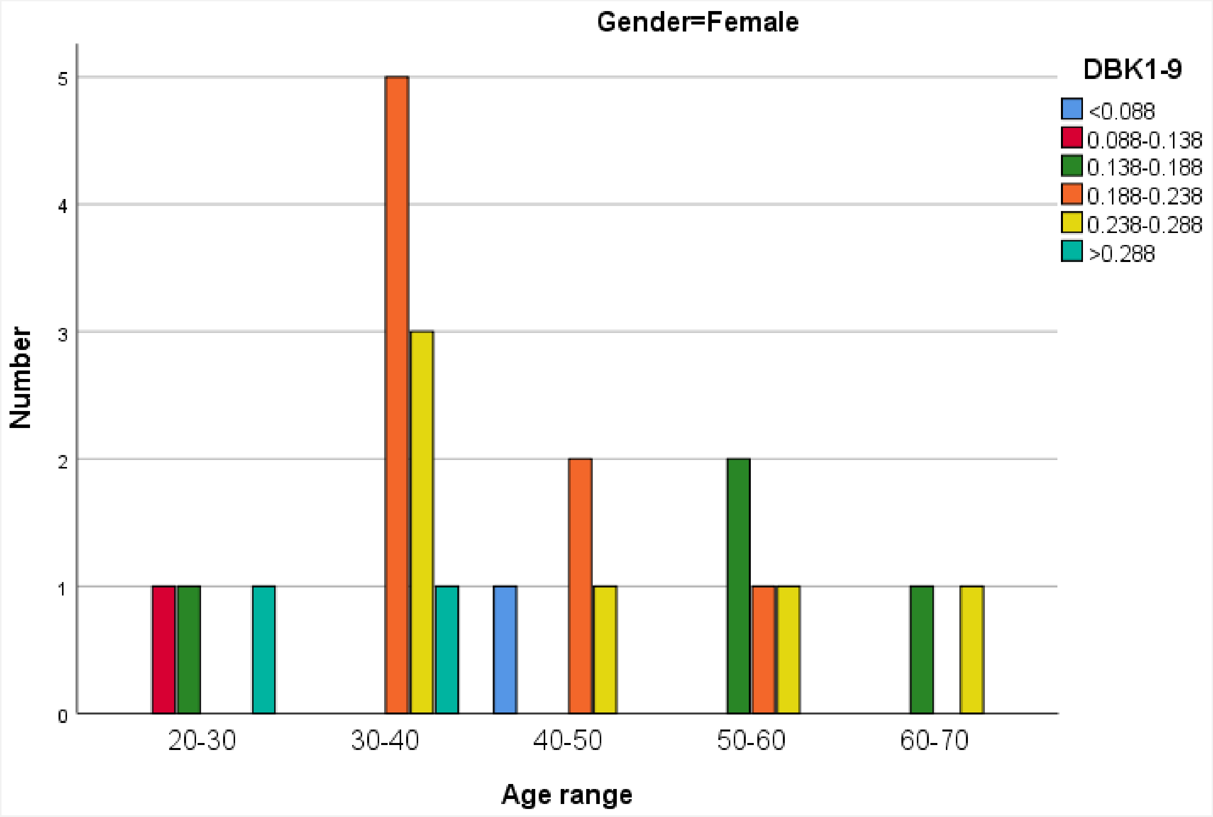

Figure S12: Gender-separated cross-tables of age and DBK1-9 ranges for the Orlando subset.

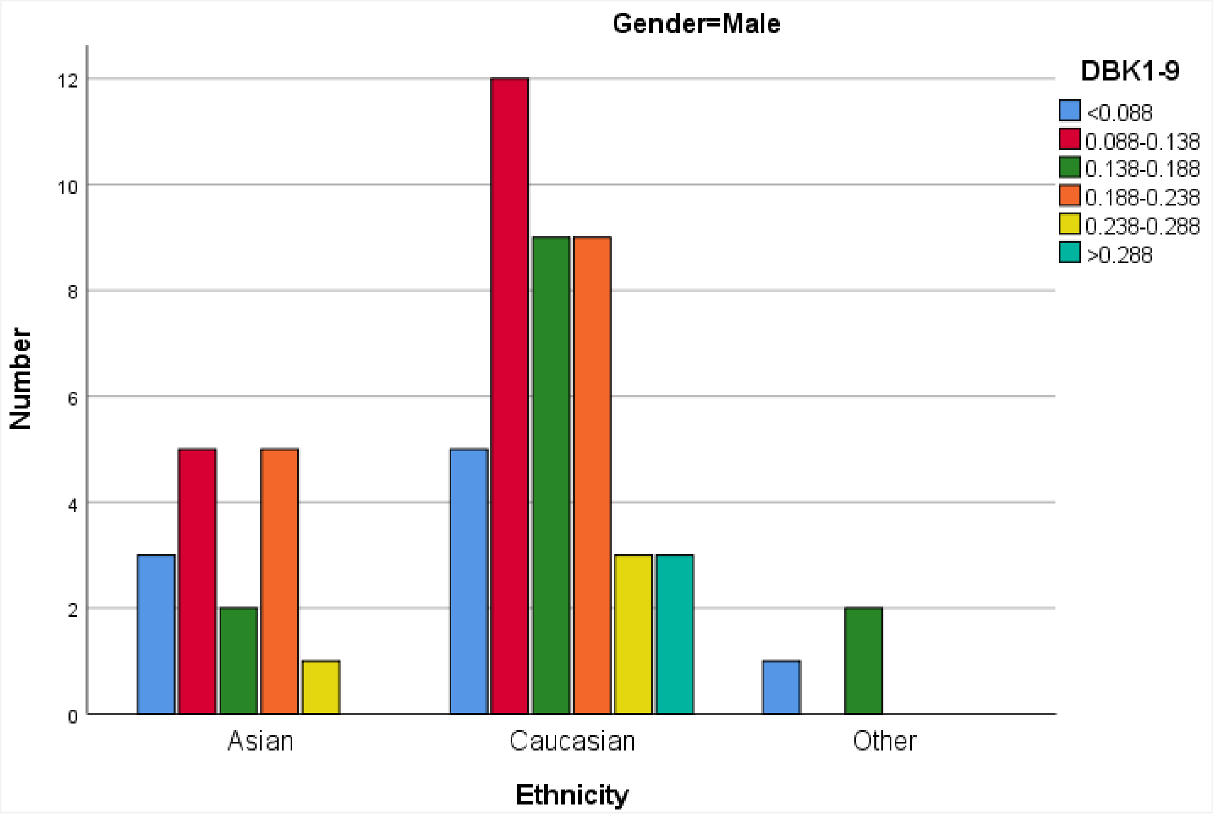

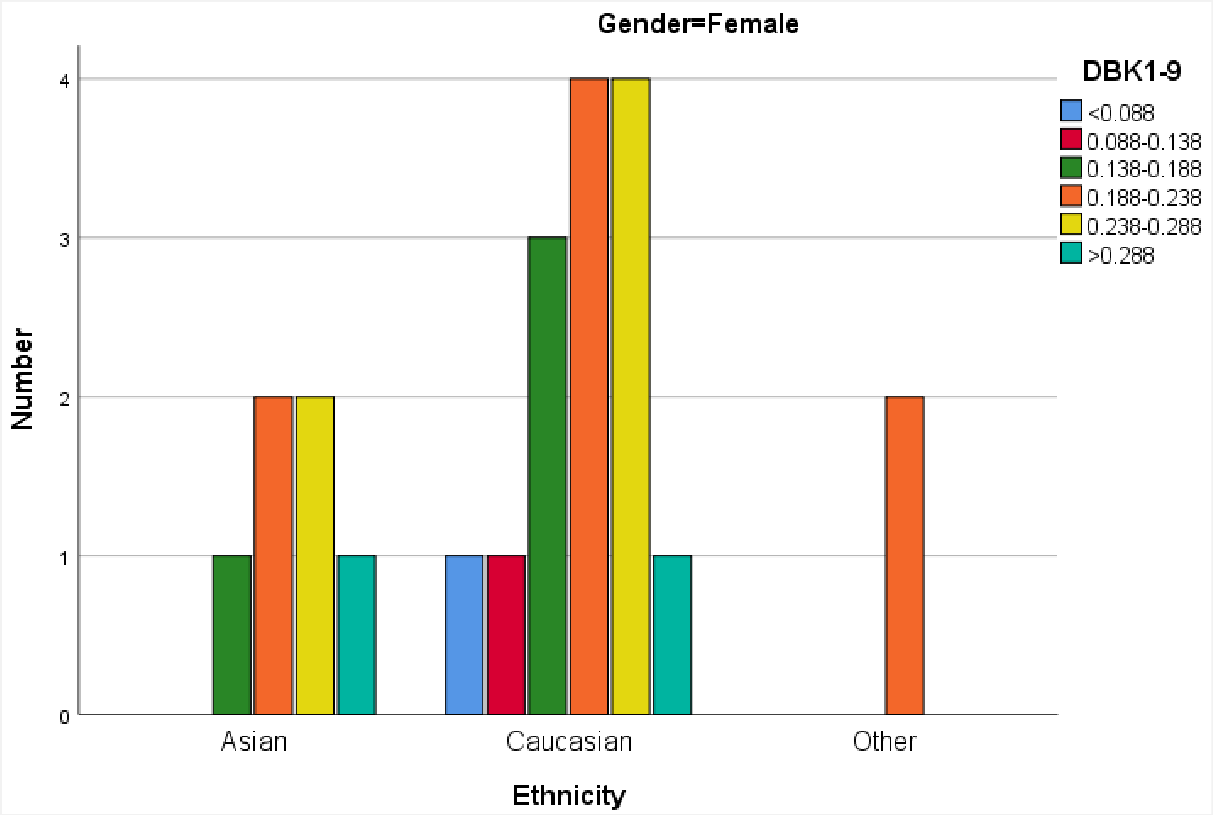

Figure S13: Gender-separated cross-tables of ethnicity and DBK1-9 ranges for the Orlando subset.

Ethnicity

Gender			Frequency	Percentage	Valid percentages	Cumulative percentages
Male	Valid	Asian	16	26,7	26,7	26,7
		Caucasian	41	68,3	68,3	95,0
		Other	3	5,0	5,0	100,0
		Total	60	100,0	100,0	
Female	Valid	Asian	6	27,3	27,3	27,3
		Caucasian	14	63,6	63,6	90,9
		Other	2	9,1	9,1	100,0
		Total	22	100,0	100,0	

Figure S14: Gender-separated sample numbers regarding ethnicity for the Orlando subset.

Age range

Gender			Frequency	Percentage	Valid percentages	Cumulative percentages
Male	Valid	20-30	7	11,7	11,7	11,7
		30-40	14	23,3	23,3	35,0
		40-50	18	30,0	30,0	65,0
		50-60	11	18,3	18,3	83,3
		60-70	8	13,3	13,3	96,7
		70+	2	3,3	3,3	100,0
		Total	60	100,0	100,0	
Female	Valid	20-30	3	13,6	13,6	13,6
		30-40	9	40,9	40,9	54,5
		40-50	4	18,2	18,2	72,7
		50-60	4	18,2	18,2	90,9
		60-70	2	9,1	9,1	100,0
		Total	22	100,0	100,0	

Figure S15: Gender-separated sample numbers regarding age for the Orlando subset.

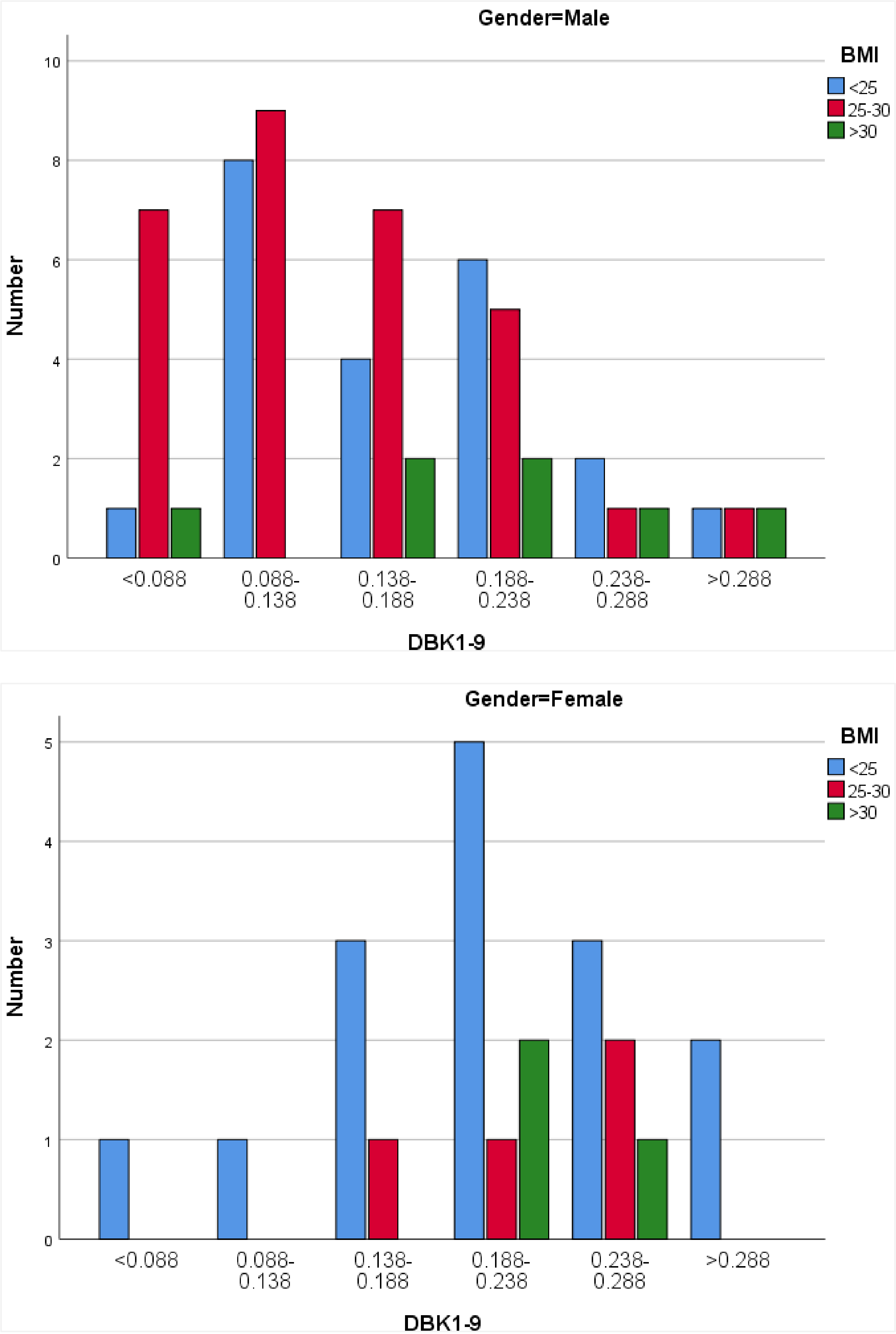

Figure S16: Gender-separated cross-tables of DKB1-9 and BMI for the Orlando subset.

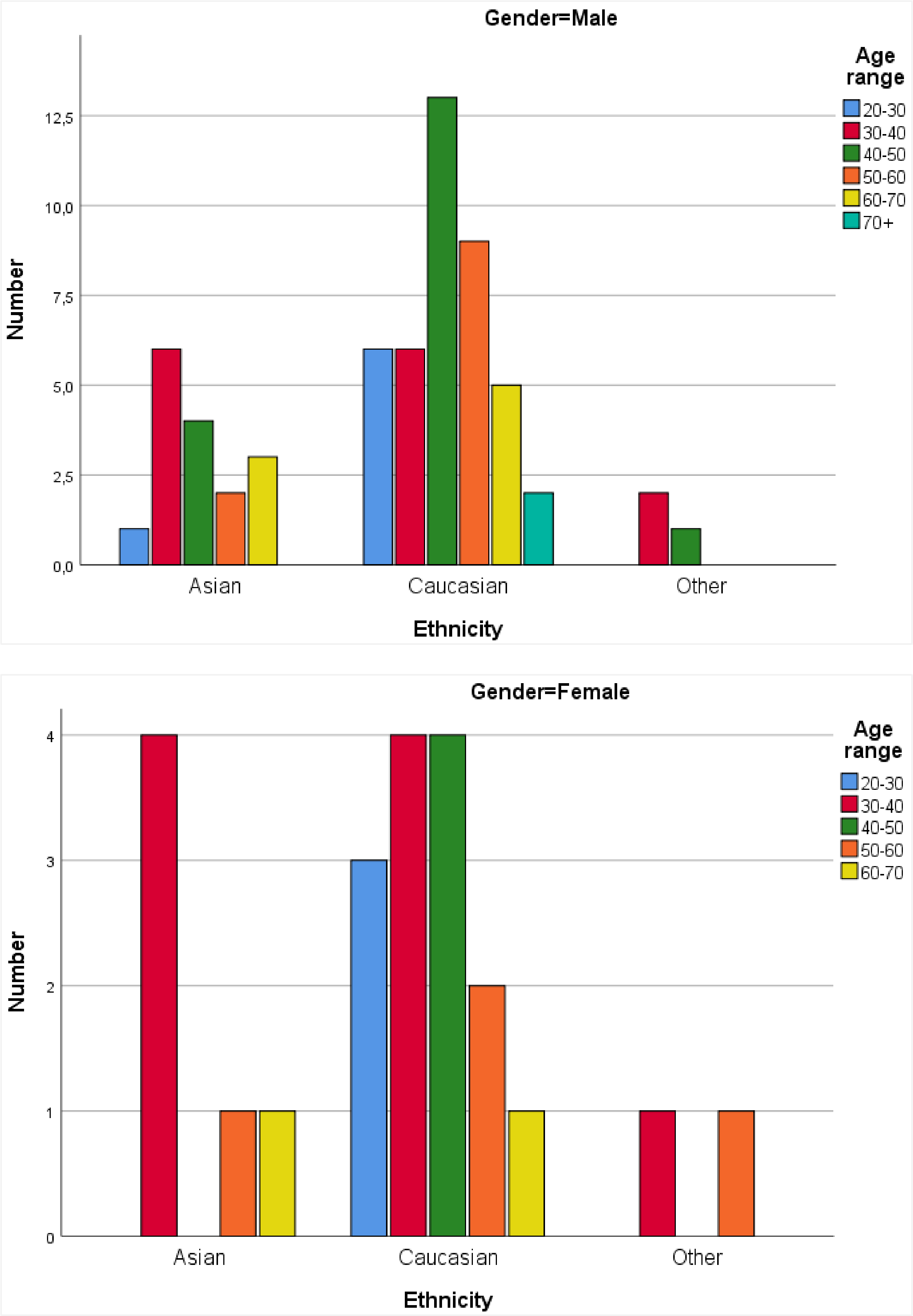

Figure S17: Gender-separated cross-tables of age and ethnicity for the Orlando subset.

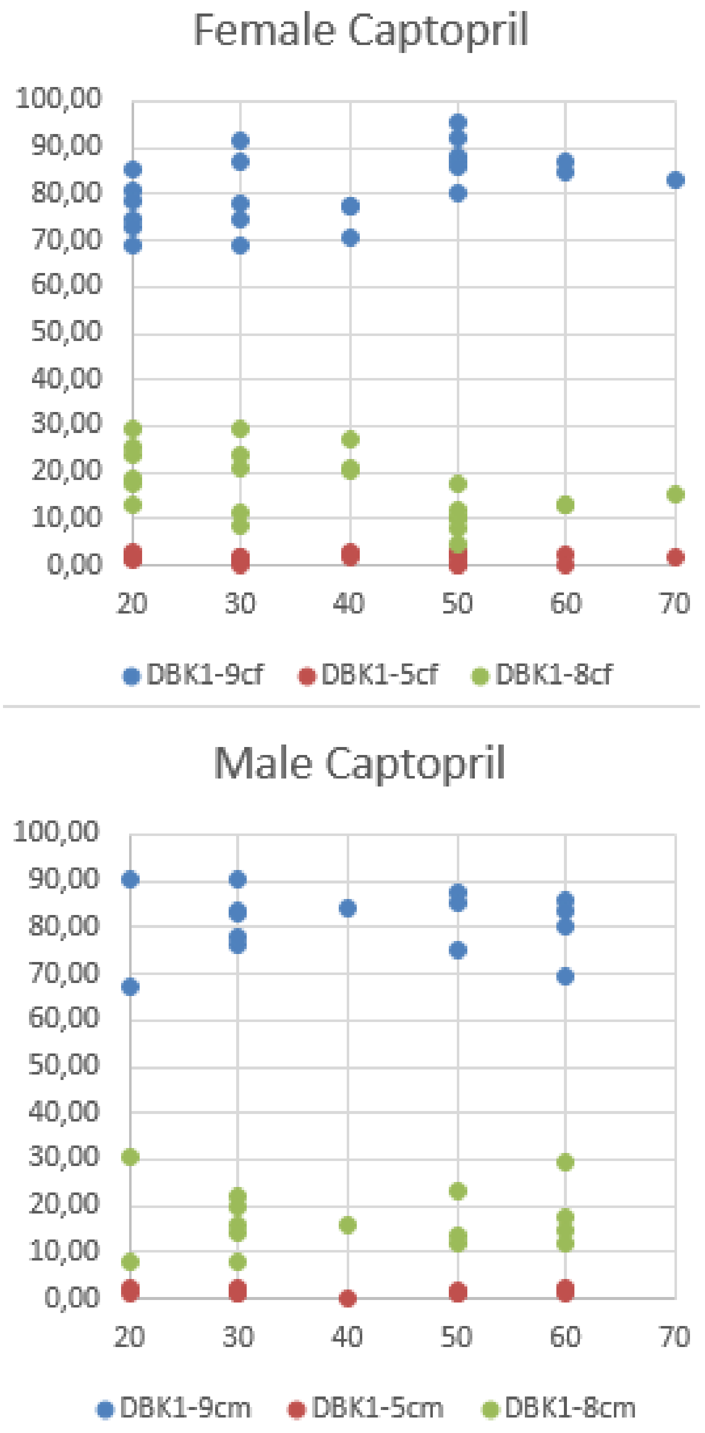

Figure S18: Inhibition with captopril of selected samples from the Taipei cohort (values in percent).
